# Ahmed Glaucoma Valve Implantation in Vitrectomized Eyes

**DOI:** 10.1155/2018/9572805

**Published:** 2018-05-10

**Authors:** Nimet Yeşim Erçalık, Serhat İmamoğlu

**Affiliations:** Haydarpaşa Numune Research and Training Hospital, Istanbul, Turkey

## Abstract

**Purpose:**

To evaluate the outcomes of Ahmed glaucoma valve (AGV) implantation in vitrectomized eyes.

**Materials and Methods:**

The medical records of 13 eyes that developed glaucoma due to emulsified silicon oil or neovascularization following pars plana vitrectomy and underwent AGV implantation were retrospectively reviewed. The main outcome measures were intraocular pressure (IOP), best-corrected visual acuity (BCVA), number of antiglaucoma medications, and postoperative complications. Surgical success was defined as last IOP ≤21 mmHg or ≥6 mmHg and without loss of light perception.

**Results:**

The mean follow-up duration was 11.7 ± 5.5 (range, 6–23) months. The mean IOP before the AGV implantation was 37.9 ± 6.7 mmHg with an average of 3.5 ± 1.2 drugs. At the final visit, the mean IOP was 15.9 ± 4.6 mmHg (*p*=0.001) and the mean number of glaucoma medications decreased to 2.3 ± 1.3 (*p*=0.021). At the last visit, 11 eyes (84.4%) had stable or improved VA and one eye (7.7%) had a final VA of no light perception. Surgical success was achieved in 11 of the 13 eyes (84.4%). Postoperative complications were bleb encapsulation (69.2%), early hypotony (38.5%), hyphema (23.1%), decompression retinopathy (23.1%), choroidal detachment (15.4%), intraocular hemorrhage (7.7%), and late endophthalmitis (7.7%). One eye (7.7%) was enucleated because of late endophthalmitis.

**Conclusions:**

Despite complications necessitating medical and surgical interventions, vitrectomized eyes were effectively managed with AGV implantation.

## 1. Introduction

Secondary glaucoma is not a rare complication following vitreoretinal surgery. It develops due to surgery and tamponading agents, is usually transient, and is generally managed with antiglaucoma therapy [1, 2]. Refractory glaucoma indicates surgical treatment, such as silicone oil (SO) removal, anterior chamber washout of emulsified SO, trabeculectomy, and valve implants [3, 4].

Vitreoretinal procedures can cause scarring and alteration of the wound healing of the conjunctiva, which can make any glaucoma surgery challenging. Furthermore, an ischemic intraocular environment due to retinal disease may deteriorate the surgical outcomes. Therefore, conventional filtering surgery in such cases has a poor prognosis. Glaucoma drainage devices (GDD) provide advantages when there is a high risk of failure with standard filtering surgery [5]. Among them, the Ahmed glaucoma valve (AGV) is easy to insert, has a wide filtration area, and prevents low IOP by functioning only when the IOP is over 8 mmHg [6, 7].

Previous studies have reported that AGV implantation has a high success rate in IOP control and a low complication rate [8, 9]. However, there are only a few studies reporting these results in the eyes that underwent vitreoretinal surgery. This study aims at evaluating the results and complications of AGV implantation in the vitrectomized eyes.

## 2. Materials and Methods

The medical records of 13 patients with a history of pars plana vitrectomy (PPV) who underwent AGV implantation between 2014 and 2016 were retrospectively reviewed. Written informed consent was obtained from each patient. The study followed the tenets of the Declaration of Helsinki.

Patients with a postoperative follow-up less than 6 months after AGV implantation, patients with a pre-AGV implantation vision of no light perception, eyes filled with SO, and eyes with a history of primary open angle glaucoma (OAG) before PPV were excluded from the study. Surgical success was defined as last IOP ≤21 mmHg or ≥6 mmHg and without loss of light perception. Failure caused by hypotony was defined as IOP of ≤5 mmHg.

Before the surgical procedure, all patients underwent an ophthalmologic examination including measurement of best-corrected visual acuity (BCVA) with the Snellen chart, biomicroscopy, gonioscopy, fundus examination, and Goldmann applanation tonometry (Haag-Streit, Köniz, Switzerland). All examinations and surgical procedures were performed by a single glaucoma specialist (SI). Demographic information, BCVA, IOP, number of glaucoma medications, history of prior vitreoretinal surgery, and postoperative complications were recorded. Postoperative visits were performed at 1 day, 1 week, 1 month, and 3 months later, and every 3 months thereafter. More frequent examinations were done when clinically necessary. After Ahmed glaucoma valve implantation, elevation of IOP was treated either medically or surgically when necessary.

### 2.1. Surgical Procedure

The AGV-FP7 model (New World Medical, Rancho Cucamonga, CA, USA) was used in all eyes. Under local or general anesthesia, the plate was implanted at the superior temporal or superior nasal quadrant by the long scleral tunnel technique. A fornix-based conjunctival flap was created at 90–120 degrees. With care to the rectus muscles, a posterior dissection was performed and the sclera was exposed for the implantation of the plate. Three scleral incisions, 10–12 mm, 6–8 mm, and 1.5–2 mm away from the limbus, respectively, were performed. The incisions, which were 2.5 mm in length and one-half to two-thirds the thickness of the sclera in depth, were made parallel to the limbus. The incisions were bonded using a 60-degree bevel-up 2.0 mm crescent knife. By bonding these three incisions, a scleral tunnel was created. An episcleral plate was inserted behind the rectus muscles and behind the equator. The plate was secured to the sclera with two absorbable 6/0 vicryl sutures. Then, the silicone tube of the device was placed in the scleral tunnel. Using the third scleral incision, parallel to the iris, a partial paracentesis was made with a 23-gauge microvitreoretinal knife. The tube was inserted 1–2 mm into the anterior chamber in 9 eyes and the ciliary sulcus in 4 eyes. The tube was shortened to prevent crystalline lens touch when necessary. The scleral incision close to the limbus was closed with an 8/0 vicryl suture to avoid leakage. The conjunctiva was sutured with 8/0 vicryl. After AGV implantation, all patients received a standard topical therapy including moxifloxacin and prednisolone for 6–8 weeks.

## 3. Statistical Analysis

Statistical analyses were performed using the IBM SPSS Statistics software (SPSS, Chicago, IL). The variables were investigated using visual (histograms, probability plots) and analytical methods (Kolmogorov–Smirnov/Shapiro–Wilk test) to determine whether or not they were normally distributed. Descriptive analyses were presented using medians and interquartile range (IQR) for the nonnormally distributed and ordinal variables. The Wilcoxon test was performed to test the significance of pairwise differences. A *p* value of less than 0.05 was considered to show a statistically significant result.

## 4. Results

Thirteen eyes of 13 patients with medically uncontrolled glaucoma after 23-gauge PPV were included in the study. The characteristics of the patients are summarized in Table 1. The mean follow-up duration was 11.7 ± 5.5 (range, 6–23) months. The mean interval between the PPV and AGV implantation was 24.1 ± 17.3 months. Indications for performing 23-gauge PPV included retinal detachment (61.5%) and neovascular glaucoma (NVG) (38.5%).

Four eyes (30.8%) had undergone glaucoma surgeries (trabeculectomy in 1 eye and cyclophotocoagulation in 3 eyes) before AGV implantation. We injected intracameral antivascular endothelial growth factor (anti-VEGF) agent, 3 days–1 week prior to the AGV implantation into 3 eyes with stage 3 of NVG (marked by secondary angle closure glaucoma) of the 5 NVG cases. One eye (7.7%) underwent combined phacoemulsification and AGV implantation.

**Figure 1:**
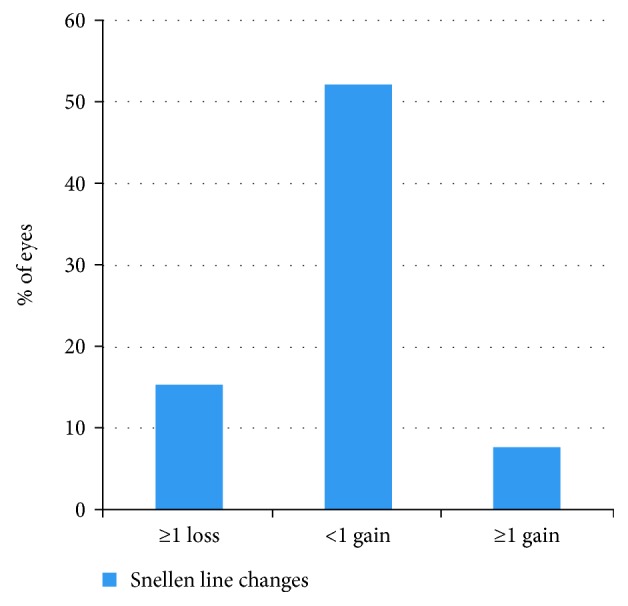
Visual gain/loss of the study patients postoperatively.

The mean IOP before the AGV implantation was 37.9 ± 6.7 mmHg with an average of 3.5 ± 1.2 drugs. At the final visit, the mean IOP was 15.9 ± 4.6 mmHg (*p*=0.001) and the mean number of glaucoma medications had decreased to 2.3 ± 1.3 (*p*=0.021). Preoperative BCVA increased from 1.58 ± 0.91 LogMAR to 1.46 ± 1.06 LogMAR at the last visit (*p*=0.7). At the final follow-up, 11 eyes (84.4%) had stable or improved VA and one eye (7.7%) had a final VA of no light perception.  Figure 1 shows the visual gain/loss of the study patients postoperatively.

We also evaluated the results separately in NVG and non-NVG cases. In the NVG group, the mean IOP before the AGV implantation was 38.4 ± 2.6 mmHg with an average of 3.8 ± 0.8 drugs. At the final visit (mean follow-up time = 11.7 months), the mean IOP was 18 ± 6.8 mmHg (*p*=0.042). We achieved the IOP control in all except one of our NVG patients following AGV implantation. We had not used anti-VEGF in that patient with uncontrolled IOP elevation. In the non-NVG group, preoperative IOP was 37.6 ± 8.6 mmHg. At the final visit (mean follow-up time = 11.7 months), the mean IOP decreased to 14.6 ± 2.2 mmHg (*p*=0.012).

**Figure 2:**
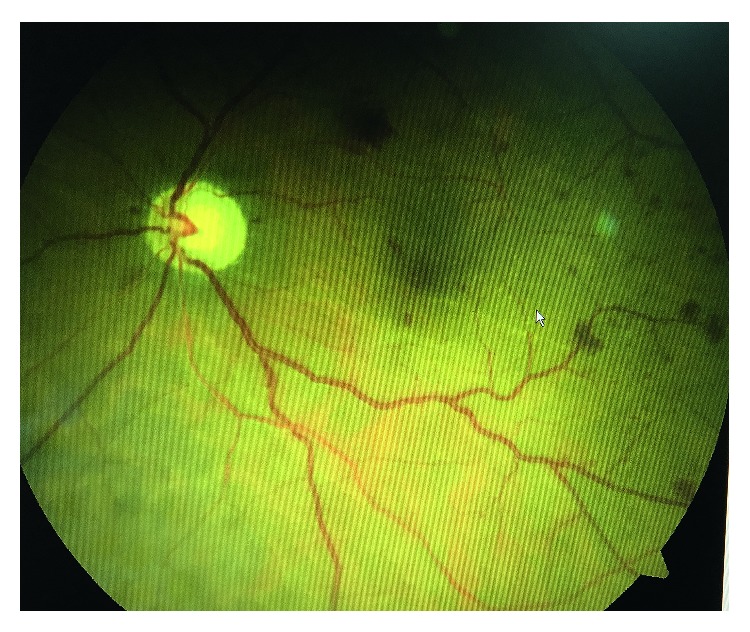
Fundus examination showing scattered retinal hemorrhages in the periphery and posterior pole typical for decompression retinopathy.

Postoperative complications were bleb encapsulation (69.2%), early hypotony (38.5%), hyphema (23.1%), decompression retinopathy (23.1%), choroidal detachment (15.4%), intraocular hemorrhage (7.7%), and late endophthalmitis (7.7%).  Figure 2 shows the fundus photo of one case with decompression retinopathy. The most commonly encountered problem was a hypertensive phase due to bleb encapsulation over the plate. Six of the 9 eyes with a hypertensive phase due to bleb encapsulation necessitated needling with 0.1 cc 5-fluorouracil (5-FU) (50 mg/ml). The mean intervention time for needling was 70.8 ± 20.2 days. One eye (7.7%) was enucleated because of late endophthalmitis. One eye (7.7%) resulted in vision with no light perception postoperatively. Except for these 2 cases, surgical success was achieved in 11 of the 13 eyes (84.6%).

## 5. Discussion

Despite several studies reporting the early transient increase in IOP after vitrectomy, limited information is available in the literature on late glaucoma after vitreoretinal surgery. Late IOP elevation after PPV was reported as 26–41% in the previous studies [10, 11].

It has been reported that PPV causes an increase in oxygen levels in the vitreous chamber that remain elevated for at least 10 months [12]. Increased oxidative damage to the trabecular meshwork [13] and small lesions created during vitrectomy leading to secondary scarring to the trabecular meshwork [14] were the two hypothesized mechanisms associated with the risk of glaucoma after PPV. In addition to these two theories, other mechanisms responsible for the IOP elevation after PPV include intraocular gas expansion, inflammation, hemorrhagic complications, silicone oil complications, pupillary block, response to steroids, and progression of the neovascularization and ciliary body edema [11, 15].

Ischemic retinal environment, conjunctival scarring, increased inflow of vasoformative factors from the vitreous cavity into the anterior chamber, and inflammation after vitreoretinal surgery were thought to result in a worse prognosis after trabeculectomy in the eyes with NVG [16, 17]. Therefore, the GDD may be preferred as the primary choice for refractory glaucoma in the eyes with previous vitreoretinal surgery. Successful long-term results were reported after AGV implantation in the eyes with NVG [18].

Inoue et al. [19] assumed that NVG may be a more significant risk factor in the vitrectomized eyes than in the nonvitrectomized eyes because vasoformative factors in the vitreous cavity could easily diffuse into the anterior chamber in the vitrectomized eyes, leading to a more severe neovascularization and inflammation. Intravitreal and intracameral bevacizumab injection have been reported to be a safe and effective adjuvant for GDD in NVG [20, 21]. In three eyes of the five cases, we used an intracameral anti-VEGF agent. We encountered hyphema in three eyes and intraocular hemorrhage in one eye, but these hemorrhages were slight and disappeared without therapy. The use of anti-VEGF agent may have prevented severe hemorrhages in our study.

It has been reported that 7.1–10% of patients who underwent PPV and SO injection developed uncontrolled glaucoma despite medical therapy and removal of SO [22]. Gupta et al. [23] reported that long-term success of AGV implantation after PPV with SO injection and subsequent removal was better than that reported for trabeculectomy. They observed 62% success at 12 months after implantation of AGV in these eyes [23]. In our series, we excluded eyes that were filled with SO and included only cases with droplets of emulsified SO. Surgical success was achieved in all of these eyes with emulsified SO, except in one eye, which was enucleated because of endophthalmitis.

The Ahmed glaucoma valve has a built-in valve mechanism which allows immediate postoperative flow and prevents hypotony but may require more glaucoma medications in the long term. We preferred to implant the AGV FP7 model in our vitrectomized eyes. A study reported that eyes with the Baerveldt tube implantation had a higher risk of refractory hypotony when compared to the eyes with AGV implantation [24]. Therefore, the AGV may be preferred for the eyes that are at risk of hypotony such as the vitrectomized eyes. However, the rate of bleb encapsulation was found to be higher in AGV compared to the Baerveldt tube in the previous studies. The use of a valve within the tube as well as the early conjunctival exposure to the proinflammatory mediators from the aqueous or the uneven surface texture of the Ahmed base plate and the small base plate area of the Ahmed valve design may be the reasons for this high encapsulation rate [25–27].

Bleb encapsulation is one of the main reasons for glaucoma valve failure [28]. Souza et al. [29] reported that previous conjunctival surgery changed the conjunctival surface, and the subsequent operation can induce a proliferation of fibrous cells leading to bleb encapsulation. It has also been reported that SO migration can occur through the tube into the subconjunctival space, causing an inflammatory reaction [30, 31]. If bleb encapsulation is encountered, needling of the bleb may help, with the aim of outflow resistance [32]. Six of the nine eyes in our study group with bleb encapsulation necessitated needling with 5-FU. In these eyes, the control of IOP was achieved in the early period following needling.

Exposure of the implant's tube with conjunctival erosion has been suggested as the main risk factor for endophthalmitis after GDD implantation [33, 34]. Also, in our case with late endophthalmitis, the conjunctival erosion was the reason for infection. We used the long scleral tunnel technique in all of the eyes in our study group. Kugu et al. [35] reported that AGV implantation performed with the long scleral tunnel technique is more effective in preventing tube exposure. They detected tube exposure in 2.5% of their patients after a mean follow-up period of 46 months.

Hypotony, hyphema, decompression retinopathy, and choroidal detachment in our patients were successfully treated with medical therapy. Intraocular hemorrhage was resolved in the early postoperative period without therapy.

Decompression retinopathy can occur after laser or medical therapy for acute glaucoma [36–38], trabeculectomy [39], and valve implantation [40, 41]. To the best of our knowledge, no study reported decompression retinopathy after AGV implantation in the vitrectomized eyes. We found the rate of decompression retinopathy as 23.1% in our series; this may be due to initial high IOP in these eyes. Operating surgeons should be aware of this complication and avoid sudden drop of IOP. Previous vitreoretinal surgery was not reported among the predisposing factors for decompression retinopathy after AGV implantation in the literature. We suspect that previous PPV may have increased the rate for decompression retinopathy in our series.

The reduction in IOP and the number of medications used postoperatively were both statistically and clinically significant in our patients. These results are consistent with previous studies evaluating AGV implantation in the vitrectomized eyes. Hong and Choi [42] found the success rate after the AGV implantation in the vitrectomized eyes as 83.4% at 6 months and 76.4% at the final visit. Preoperative IOP decreased from 47.5 mmHg with 1.76 drugs to 13.8 mmHg with 0.35 drug at 6 months. Park et al. [43] reported the success rate of AGV implantation as 83.8% at 1 year in their vitrectomized patients. Final visual acuity improved or stabilized in 78.6% of their cases, which is consistent with our results. Cheng et al. [44] evaluated the effect of AGV implantation in diabetic vitrectomized eyes. Postoperatively, IOP decreased from 49.4 mmHg to 17.5 mmHg. Sixty-six point seven percent of the eyes still needed average 0.8 drug.

Limitations of this study are retrospective design, small sample size, short follow-up time, variation of the etiologic factors for PPV, and the lack of a control group. Despite complications necessitating medical and surgical interventions, vitrectomized eyes were effectively managed with AGV implantation. A larger series with a longer follow-up is required.

## Figures and Tables

**Table 1:** Characteristics of the study patients.

Age (years)	53.93 ± 16 (23–78)
Gender (female/male), number (%)	3 (23.1), 10 (76.9)
Preoperative BCVA (LogMAR)	1.58 ± 0.91
Preoperative IOP (mmHg)	37.9 ± 6.7
Preoperative number of glaucoma medications	3.5 ± 1.2
Lens status (eyes/%)	
Phakia	2 (15.4)
Pseudophakia	9 (69.2)
Aphakia	2 (15.4)

Intraocular pressure (IOP); best-corrected visual acuity (BCVA).
